# Deep Reinforcement Learning for Attacking Wireless Sensor Networks

**DOI:** 10.3390/s21124060

**Published:** 2021-06-12

**Authors:** Juan Parras, Maximilian Hüttenrauch, Santiago Zazo, Gerhard Neumann

**Affiliations:** 1 Information Processing and Telecommunications Center, Universidad Politécnica de Madrid, 28040 Madrid, Spain; santiago.zazo@upm.es; 2 Lincoln School of Computer Science, University of Lincoln, Lincoln LN6 7TS, UK; maximilian.huettenrauch@kit.edu (M.H.); gerhard.neumann@kit.edu (G.N.); 3 Karlsruhe Institute of Technology, 76131 Karlsruhe, Germany

**Keywords:** POMDP, Deep Reinforcement Learning, TRPO, SSDF attack, backoff attack

## Abstract

Recent advances in Deep Reinforcement Learning allow solving increasingly complex problems. In this work, we show how current defense mechanisms in Wireless Sensor Networks are vulnerable to attacks that use these advances. We use a Deep Reinforcement Learning attacker architecture that allows having one or more attacking agents that can learn to attack using only partial observations. Then, we subject our architecture to a test-bench consisting of two defense mechanisms against a distributed spectrum sensing attack and a backoff attack. Our simulations show that our attacker learns to exploit these systems without having a priori information about the defense mechanism used nor its concrete parameters. Since our attacker requires minimal hyper-parameter tuning, scales with the number of attackers, and learns only by interacting with the defense mechanism, it poses a significant threat to current defense procedures.

## 1. Introduction

Reinforcement learning (RL) is a field that has attracted great attention in the last twenty years in the community of artificial intelligence. It allows programming agents to learn by interacting with a complex, dynamic environment: the agent observes its state, chooses actions, obtains a reward and transitions to another state. It is important to remark that RL does not require indicating how the agent is to act; instead, the agent learns to choose its action by trial and error, such that its total reward is maximized. Introductory material for RL can be found in [1,2].

The recent advances in the field of Deep Learning [3] have produced a massive step forward in the RL field. A seminal work was the publication of the Deep Q-Networks (DQN) algorithm [4,5], which offered superhuman performance in a set of Atari games. Several other algorithms for Deep RL (DRL) followed and were applied successfully to complex problems [6,7,8,9,10].

These advances do affect many problems of interest in the area of Wireless Sensor Networks (WSNs). A survey notes that RL algorithms are used in several problems that arise in WSNs, such as routing, data latency, path determination, duty cycle management, QoS provisioning or resource management [11]. Another problem is the security in WSNs, where a great deal of research is being done at the moment [12,13,14,15,16], also taking advantage of these recent advances in deep learning [17,18]. The idea of applying RL to cyber security is not new [19,20]; to mention some examples, DRL tools are used in WSN security to detect spoofing attacks [21], for mobile off-loading [22,23], to avoid jamming [24,25] and to model DoS attacks [26] or data poisoning attacks [27].

However, many current defense mechanisms designed for WSNs are ad hoc, that is, designed against a specific attack [28,29,30]. However, the advances in DRL potentially open the door to designing an attacker that learns to exploit a possibly unknown defense mechanism, as these methods follow a trial-and-error approach to learn an optimal behavior. Inspired by these advances, we apply DRL techniques for learning an optimal attack against WSN defense mechanisms that are unknown to the attacker. Thus, the question we pose is, given a WSN in which there are several Good Sensors (GSs) and one or more Attacking Sensors (ASs), how do current defense mechanisms perform against DRL-based attackers? Can we learn an attacker using DRL that exploits unknown defense mechanisms simply by interacting with them?

A previous work [31] showed that DRL techniques could be used to obtain quasi-optimal attacks in a Cooperative Spectrum Sensing network using a robust defense mechanism. This paper expands that work significantly, as we are interested in the multiple ASs case. Moreover, we study whether the ASs pose a larger threat to the defense mechanism if the ASs are able to communicate and cooperate on the task of attacking the defense mechanism. Compared to [31], our approach has several important differences. First, we do not assume that the state is observable for the ASs: they will only have partial information about the system, which is a far more realistic setting. This means that the ASs do not have access to the precise state of the defense mechanism (e.g., the reputation of each node), as they only have access to values that can be observed, such as the times when a node attempts to transmit. Second, we study two different defense mechanisms on the PHY and MAC layers: in the former, the action space is continuous, while in the latter, the action space is discrete. Our Deep Learning Attacker (DLA) is able to work with both types of action spaces, thus giving it more flexibility. Third, we focus on the case in which there are more than one AS, enabling the ASs to communicate their observations to other ASs in order to better exploit the defense mechanism. Since all the ASs have a common goal, which is to exploit the defense mechanism of the WSN, the ASs can be studied as a swarm [32], which is a group of homogeneous agents that cooperate to obtain a common goal in a decentralized fashion. While there is a large body of research devoted to the optimization of swarms from the evolutionary perspective, including [33,34,35,36], we use gradient-based optimization methods as in [37].

Our results show that our DLA architecture does pose a strong challenge to WSN security that current ad-hoc defense mechanisms fail to deal with. The threat that DRL methods pose to current defense mechanisms when ASs can communicate among them is surprisingly not well assessed in the current literature to the best of our knowledge.

This paper is structured as follows. We start by describing the mathematical foundations that our work is based on in Section 2. Section 3 introduces the defense mechanisms for the two attacks we study. Afterwards, Section 4 and Section 5 introduce our DLA architecture and validate our approach via simulations, in which we test the effectiveness of our DLA. Finally, in Section 6, we discuss the results and draw some conclusions.

## 2. Background

We now introduce several key concepts to understand our model. We start by introducing Markov Decision Processes (MDPs), Partially Observable MDPs (POMDPs) and a swarm model. We also explain a DRL algorithm, Trust Region Policy Optimization (TRPO).

### 2.1. Deep Reinforcement Learning

Let us start by considering that there is a single AS. MDPs are a common framework to study optimal control problems and can be defined as [38]:

**Definition** **1.** *An MDP is a 5-tuple 〈S,A,P,R,γ〉, where:*

- *S is a finite set of states s.*
- *A is a finite set of actions a.*
- *$P_{a}\left(s^{n},s^{n+1}\right)$ is the probability that action a in state $s^{n}$ and time step n will lead to state $s^{n+1}$ in time step n+1.*
- *$R_{a}\left(s^{n},s^{n+1}\right)$ is the reward received after transitioning from state $s^{n}$ to state $s^{n+1}$ due to action a.*
- *γ is a discount factor.*

An MDP policy π is a mapping π:S→A that associates an action (or a probability distribution over actions) to each state. We can define π as a function of the current state because the Markov property holds: the probability to reach state *s* in time *n* depends only on the state in time n−1.

There are several ways of solving an MDP. One approach is using Dynamic Programming methods [39], which require knowing $P_{a}$. Another approach is RL, which obtains an optimal policy without explicitly knowing the transition function $P_{a}$. There are several RL algorithms that could be used, such as Q-learning or SARSA [1]. However, these RL algorithms are impractical when the state and/or the action space are large or continuous. For these cases, there has been recently a lot of research that uses the advances in Deep Neural Networks (DNNs) to propose DRL algorithms, which are able to cope with continuous, large state spaces, such as DQN [4,5] or DRQN [6], and even with continuous action spaces, such as TRPO [9] or PPO [10].

We use TRPO [9] as a DRL algorithm in this work. TRPO uses a DNN of parameters θ to approximate the policy $\pi_{\theta }$. The policy is updated by solving the following optimization problem:

(1) $$\begin{array}{cc} \underset{\theta }{\mathrm{maximize}} & \;E\left[\frac{\pi_{\theta }}{\pi_{\mathrm{old}}}A\left(s^{n},a\right)\right]\\ \mathrm{subject}\;\mathrm{to} & \;E\left[D_{\mathrm{KL}}(\pi_{\theta }\left|\right|\pi_{\mathrm{old}})\right]\leq \delta , \end{array}$$

where E is used to denote the mathematical expectation, $\pi_{\mathrm{old}}$ refers to the policy in the previous iteration, $A(s^{n},a)$ is the advantage function, which gives an estimate of how good in terms of reward it is to use action *a* in state $s^{n}$. $D_{\mathrm{KL}}$ denotes the Kullback–Leibler divergence, and δ is a constant that forces the new policy to be closer than δ to the old policy.

The rationale for using TRPO is twofold. Firstly, it allows working with continuous and discrete action spaces [9], which provides flexibility to our DLA. Furthermore, TRPO is a powerful DRL algorithm, which has shown to benchmark very well in different sets of tasks [40].

### 2.2. Partially Observable Markov Decision Processes

In our case, the real state of the network is typically unknown and the agents can only obtain local observations such as their last actions and rewards. Thus, we need to introduce the concept of POMDP, which generalizes the MDP framework. A POMDP is defined as follows [38]:

**Definition** **2.** *A POMDP is a 7-tuple*

*〈S,A,P,R,γ,Ω,O〉, where:*

- *S, A, $P_{a}\left(s^{n},s^{n+1}\right)$, $R_{a}\left(s^{n},s^{n+1}\right)$ and γ are defined as in the MDP case.*
- Ω *is a finite set of observations o.*
- *$O_{a}\left(o^{n},s^{n}\right)$ is the probability of observing $o^{n}$ given that the actual state is $s^{n}$ and the agent takes action a.*

In an MDP, the observation corresponds to the state. For POMDPs, the state is not directly observable and hence the Markovian assumption does not hold: choosing an optimal action in time *n* in general requires knowing the whole history of past observations $o^{n}$ for n∈{0,1,...,n}; thus POMDPs are computationally complex to solve. If *N* and the sets *S*, *A* and *O* are finite, there exist algorithms that can obtain the optimal policy [38]. Another way of solving POMDPs consists in obtaining a belief, which is a sufficient statistic that collects all the pertinent past information and allows turning the POMDP in an MDP over the belief space [38]. An alternative way of solving POMDPs is based on Predictive State Representation (PSR) [41,42]. Finally, there are methods that use DRL tools to obtain approximate solutions for POMDPs, which can be classified in two main branches according to [6]:

1. Using Recurrent Neural Networks (RNNs), which are able to store past information. This solution is proposed to learn POMDPs in [6].
2. Using a finite vector of past observations as input to the policy DNN. As [6] shows, this solution is an approximation for solving POMDPs, but it can provide very good results [5] while keeping a lower computational complexity. This motivates us to use this approach in this paper.

### 2.3. The Swarm Model

In this paper, we want to model more than one AS and study a potential performance increase if the ASs are able to communicate. If we have more than one AS, it is important to note that all ASs will share a common goal, which in this case is to exploit the defense mechanism of the network. Our case shares a lot of similarities with the swarm robotics literature [37]. A swarm is a group of homogeneous agents that cooperate to obtain a common goal in a decentralized fashion. In our case, the ASs need to cooperatively attack the defense mechanism. There is extensive ongoing research that tries to optimize the behavior of a swarm from an evolutionary perspective, taking inspiration from nature [36]. Some examples of these algorithms are Krill Herd algorithm [33,43], Cuckoo Search [44,45], Monarch Butterfly Optimization [35,46] or Elephant Herding Optimization [34,47]. Instead of using these models, we use a gradient-based approach as in [37] to train our policy DNN.

There are several ways of modeling a swarm from a dynamic systems perspective. A frequent model is the Dec-POMDP framework [48,49], Ch. 15, which, unfortunately, has a NEXP-Complete complexity in the worst case [50]. This problem affects even recent algorithms such as [51], which can be applied only in very limited time horizons. In order to alleviate the complexity that arises under the Dec-POMDP model, a particularization of this framework for swarms was proposed in [32], called the swarMDP:

**Definition** **3.** *A swarMDP is defined in two steps. First, we define A=〈S,A,Ω,π〉, where the agent prototype A is an instance of each agent of the swarm:*

- *S is the set of local states.*
- *A is the set of local actions.*
- Ω *is the set of local observations.*
- *π is the local policy.*

*A swarMDP is a 7-tuple 〈I,A,P,R,O,γ〉 where:*

- *I is the index set of the agents, where i=1,2,...,N indexes the agent.*
- *A is the agent prototype defined before.*
- *$P_{a}\left(s^{n},s^{n+1}\right)$ is the transition probability function defined as in the POMDP. P depends on a, the joint action vector and s is the global state.*
- *$R_{a}\left(s^{n},s^{n+1}\right)$ is the reward function defined as in the POMDP case. R depends on the joint action vector a. In addition, all agents i share the same reward function.*
- *$O_{a}\left(o^{n},s^{n}\right)$ is the observation model, defined as in the POMDP case. O depends on the joint action and observation vectors a and o, respectively.*
- *γ∈[0,1] is a discount factor as in the POMDP case.*

The main difference between the swarMDP and the Dec-POMDP model lies in the fact that the swarMDP explicitly assumes that all agents are homogeneous. Whereas under the Dec-POMDP model, each agent could have a different action and/or observation set, under the swarMDP model, all agents share the same local state space, action space, observation space and policy. Due to this characteristic, which is called the homogeneity property, the agents are interchangeable. In addition, a single agent swarMDP reduces to a POMDP.

The homogeneity property simplifies the problem of learning, i.e., the order of the agents does not matter. Note that all agents share the same policy due to the homogeneity property (i.e., each agent would act like the others if they observed the same observation $o^{n}$). Thus, we can use single-agent DRL algorithms and a centralized training/decentralized execution method to find a policy [37]. In our case, we make use of TRPO and train a single policy $\pi_{\theta }$ for all agents, which takes as input the local observation of each agent *o* and outputs a local action *a*. During training, the local observations of each agent are sent to the central learning algorithm for training. During execution, each agent uses a copy of the learned policy.

Finally, the observation vector of agent *i* may include not only information about agent *i* but also information about other agents if the agents are able to communicate. Let us denote by $o_{i,i}^{n}$ the information available to agent *i* about itself in time *n*, and $o_{i,j}^{n}$ the information available to agent *i* about agent *j*, j≠i. A naive way of encoding this information is to build the total observation vector of agent *i*, $o_{i}^{n}$ by simply concatenating $o_{i,i}^{n}$ and all the vectors $o_{i,j}^{n}$. However, this concatenation causes a large input space, as well as ignoring the permutation invariance inherent to a homogeneous swarm. A better option consists in using mean embeddings [37,52], which are based on the fact that a probability distribution P(X) can be represented as an element in a reproducing kernel Hilbert space by its expected feature map $\mu_{X}=E_{X}\left[\phi \left(X\right)\right]$, where ϕ(X) is a feature mapping. There are several possible mean embeddings that can be used, depending on the feature mapping choice. We choose to employ the following:

- Neural Networks Mean Embedding (NNME): In this approach, each $o_{i,j}^{n}$ is used as input to a DNN, which outputs $\phi (o_{i,j}^{n})$, where ϕ denotes the transformation done by the DNN. The total observation vector of agent *i*, $o_{i}^{n}$ is built by concatenating $o_{i,i}^{n}$ to the mean of the set of all $\phi (o_{i,j}^{n})$.
- Mean-based Mean Embeddings (MME): Under this approach, we average $o_{i,j}^{n}$ and concatenate it to $o_{i,i}^{n}$. This vector is the input to the policy network.

Thus, mean embeddings are thus insensitive to the number of agents *j* and are also insensitive to their order. The NNME is trained at the same time as the policy network, allowing NNME to adapt to a concrete problem setup.

## 3. Defense Systems

We describe two defense mechanisms based on [30] for two different attack scenarios in a WSN: a Spectrum Sensing Data Falsification (SSDF) attack and a backoff attack. The former is a physical layer defense mechanism, while the latter is an MAC layer defense mechanism.

### 3.1. Spectrum Sensing Data Falsification Attack

The SSDF is an attack that affects the physical layer, addressed against Cooperative Spectrum Sensing (CSS), in which a WSN is used to determine whether a wireless channel is being used or not. A survey of different SSDF attacks can be found in [53].

**Figure 1:**
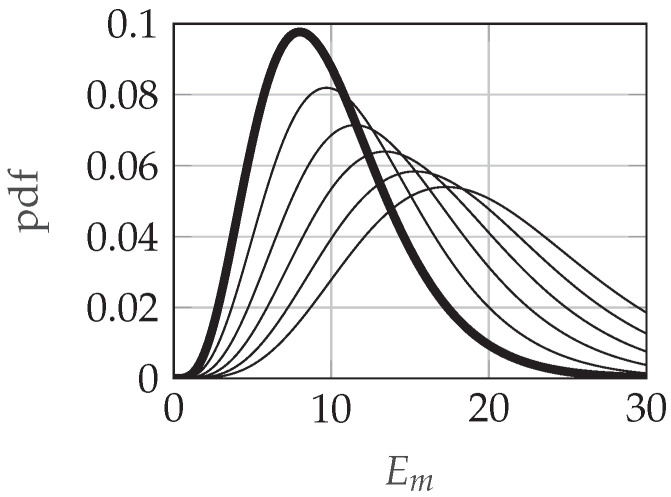
Illustration of the pdf of the chi-squared distributions from (2). The thick pdf corresponds to the $H_{0}$ case: the chi-squared $\chi_{2k}^{2}$ distribution; and the thinner pdfs correspond to the $H_{1}$ case: the non-central chi-squared $\chi_{2k}^{2}\left(2SNR_{m}\right)$ distributions for SNR values {2,4,6,8,10}, from left to right in the plot. For all curves, k=5 is the time-bandwidth parameter. Observe that, as the SNR increases, the pdf curves are more separated for $H_{0}$ and $H_{1}$.

We use a defense mechanism based on soft fusion: the reports sent by the sensors to a central entity known as Fusion Center (FC) are the energy levels $E_{m}$ they sense. As shown by [54], if the channel is idle (i.e., only noise is in the channel), $E_{m}$ follows a chi-square distribution, whereas if a signal is present, $E_{m}$ follows a non-central chi-square distribution. We use a hypothesis test, where $H_{0}$ means that the channel is idle and $H_{1}$ means that the channel is busy, and hence:

(2) $$E_{m}∼\left\{\begin{array}{ccc} \chi_{2k}^{2} & \mathrm{if} & H_{0}\\ \chi_{2k}^{2}\left(2SNR_{m}\right) & \mathrm{if} & H_{1} \end{array}\right.,$$

where *k* is the time-bandwidth product and $SNR_{m}$ is the signal-to-noise ratio in sensor *m* in natural units. An illustration of these probability density functions (pdfs) can be found in Figure 1.

The SSDF attack proposed in [30] consists in reporting honestly $E_{m}$ if $E_{m}>\xi$ and $E_{m}+\Delta$ if $E_{m}\leq \xi$, where Δ and ξ are attack parameters: Δ is the bias in the energy level introduced by the AS and ξ is the attack threshold. In other words, the AS reports that the channel is busy when it is actually idle if a certain threshold in the energy level is satisfied. We set Δ by using the means of the distributions (2), where the mean values μ of the distributions under $H_{0}$ and $H_{1}$ are:

(3) $$\mu_{0}=2k,\;\;\mu_{1}=2k+2SNR_{m}.$$

We set $\Delta =\mu_{1}-\mu_{0}=2SNR_{m}$, resulting in a defense mechanism that is tuned to detect a bias that tries to simulate the $E_{m}$ values when the channel is busy. Graphically, according to Figure 1, this Δ value translates the $H_{0}$ pdf to the right Δ units. Depending on the value of ξ, $E_{m}$ values that are actually produced under $H_{1}$ are also translated and some $E_{m}$ measurements produced under $H_{0}$ are not. Observe that if the pdfs under $H_{0}$ and $H_{1}$ are close and overlap significantly (i.e., $SNR_{m}$ is low for sensor *m*), attacking may be unnecessary in many cases.

We denote by $G_{0}$ the situation in which an AS does not attack and $G_{1}$ when it attacks. Under attack, the pdf from (2) can be written as:

(4) $$E_{m}∼\left\{\begin{array}{ccc} \chi_{2k}^{2} & \mathrm{if} & G_{0},H_{0}\\ \chi_{2k}^{2}\left(2SNR_{m}\right) & \mathrm{if} & G_{0},H_{1}\\ \chi_{2k}^{2}+\Delta & \mathrm{if} & G_{1},H_{0}\\ \chi_{2k}^{2}\left(2SNR_{m}\right) & \mathrm{if} & G_{1},H_{1} \end{array}\right.,$$

where we approximate the situation $G_{1},H_{0}$ by a translation of the chi-squared pdf when $G_{0},H_{0}$. The accuracy of the approximation depends on the threshold ξ value. In addition, observe that (4) assumes that under $H_{1}$ hypothesis, there is no attack: again, this assumption is an approximation that depends on ξ.

The defense mechanism proposed in [30] is based on two Neyman-Pearson tests, which we summarize here for a better understanding. The first test decides whether sensor *m* senses a busy channel using reports from other sensors as:

(5) $$\prod_{i=1,i\neq m}^{M}\frac{P(E_{i}=e_{i}|\chi_{2k}^{2}\left(2SNR_{m}\right))}{P(E_{i}=e_{i}|\chi_{2k}^{2})}\underset{H_{0}}{\overset{H_{1}}{≷}}\eta ,$$

where $H_{1}$ and $H_{0}$ are the energies from (2) and η is the threshold of test 1.

The second test is used to individually detect which sensors are providing false reports (i.e., the sensors that are attacking). This test is only used for sensor *m* if $H_{0}$ was the result of test 1 (i.e., only noise detected) using the expressions from (4) as:

(6) $$\frac{P(E_{m}=e_{m}-\Delta |\chi_{2k}^{2})}{P(E_{m}=e_{m}|\chi_{2k}^{2})}\underset{G_{0}}{\overset{G_{1}}{≷}}\zeta ,$$

where ζ is the threshold of test 2.

**Figure 2:**
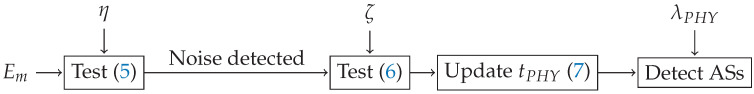
Block diagram for the defense mechanism against the SSDF attack. The inputs are the energy levels reported by the sensors $E_{m}$, the defense mechanism parameters are η, ζ and $\lambda_{PHY}$, and the result is an updated list of sensors detected as ASs.

Test 2 allows detecting whether a sensor *m* is attacking or not. The defense mechanism keeps a reputation scheme, in which there are two values *r* and *s* for each sensor in the WSN, where *s* keeps track of how many times sensor *m* has attacked (*s*) and *r* counts how many times sensor *m* has not attacked. The reputation of each sensor $t_{PHY}$ is computed as [30]:

(7) $$t_{PHY}=\frac{r+1}{r+s+2},$$

where, if $t_{PHY}$ falls below a certain threshold $\lambda_{PHY}$, the sensor is considered to be an AS and is banned from the network. While in [30] the attack policy was fixed beforehand, we let our DLA choose the energy level $E_{m}$. Thus, it has to learn which value of $E_{m}$ it should send and adapt it dynamically in order not to be detected by the defense mechanism. A scheme summarizing the defense mechanism is in Figure 2.

### 3.2. Backoff Attack

The second defense mechanism used in our experiments is a backoff attack, which affects the MAC layer of any protocol that uses CSMA/CA mechanism (such as IEEE 802.11, ref. [55] and most WSN proposed MAC protocols [56,57]). If the attack is successful, the ASs reach a higher share of the network throughput by not respecting the backoff procedure. A study of its effects can be found in [58].

As a defense method, we use a modified Cramer-von Mises (CM) statistical test [59] as in [30]. The CM test is fast to compute and allows deciding whether a stream of data is adjusting to a certain distribution, using the cumulative distribution function (CDF). We denote by $x_{m}$ the observed backoff time, which is the estimated backoff window size that sensor *m* has used and is observed by the FC.

There are several parameters that need to be known in order to obtain $x_{m}$. According to the 802.11 standard [55], using the BA (Basic Access) mechanism, we can obtain the time that the channel is occupied when there is a transmission ($T_{t}$) or a collision ($T_{c}$) as [60]:

(8) $$\left\{\begin{array}{c} T_{c}=H+T_{p}+DIFS+\delta \\ T_{t}=H+T_{p}+SIFS+T_{\delta }+ACK+DIFS+T_{\delta } \end{array}\right.,$$

where *H* is the total header transmission time (adding PHY and MAC layers headers), DIFS and SIFS are interframe spacing defined in the standard, ACK is the transmission time of an acknowledgement frame (ACK), $T_{\delta }$ is the propagation delay and $T_{p}$ is the time used to transmit payload bits. In this work, we use the values in Table 1. Using these values, the FC can obtain $x_{m}$ as shown in [30].

In order to model the real distribution of the window backoff, we follow the mechanism used in [55]. After a successful transmission, the backoff window size is divided by 2, whereas after a collision, the backoff window size is doubled. The backoff window size is forced to be in the interval $\left[CW_{min},CW_{max}\right]$, where these parameters are the minimum and maximum backoff window size, respectively. We consider that $CW_{min}=32=2^{5}$ and $CW_{max}=1024=2^{10}$ as in [55], with $p_{c}$ being the collision probability, $n_{c}$ the number of collisions and U[α,β] the random integer uniform distribution between α and β; hence, the distribution of the window backoff if there is no attack, $f_{o}\left(x_{m}\right)$, is

(9) $$f_{0}\left(x_{m}\right)=\left\{\begin{array}{ccccc} \sum_{j=0}^{n_{c}}U\left[0,2^{5+j}\right] & w.p. & p_{c}^{n_{c}}\left(1-p_{c}\right) & \mathrm{if} & n_{c}\leq 5\\ \sum_{j=0}^{5}U\left[0,2^{5+j}\right]+\sum_{j=6}^{n_{c}}U\left[0,2^{10}\right] & w.p. & p_{c}^{n_{c}}\left(1-p_{c}\right) & \mathrm{if} & n_{c}>5 \end{array}\right.,$$

where w.p. stands for with probability. The collision probability $p_{c}$ can be estimated by the FC by counting the number of successful transmissions and the number of collisions, and computing the proportion of collisions. Using (9) and the estimated $p_{c}$, we can obtain $F_{0}\left(x_{m}\right)$, the cumulative distribution of $f_{0}\left(x_{m}\right)$.

The modified CM test proposed in [30], which we summarize here for the sake of clarity, requires *K* observations $x_{1},x_{2},...,x_{K}$ from sensor *m*, which are used to obtain $F_{1}$, the empirical CDF of the window size from sensor *m*. The test also requires *L* samples $y_{1},y_{2},...,y_{L}$ generated from the real distribution when there is no attack. The test statistic for each sensor θ is obtained as

(10) $$\begin{array}{cc} \theta_{1} & =\sum_{j=1}^{K}\mathrm{sgn}\left(F_{0}\left(x_{m}\right)-F_{1}\left(x_{m}\right)\right){\left[F_{0}\left(x_{m}\right)-F_{1}\left(x_{m}\right)\right]}^{2},\\ \theta_{2} & =\sum_{j=1}^{L}\mathrm{sgn}\left(F_{0}\left(y_{m}\right)-F_{1}\left(y_{m}\right)\right){\left[F_{0}\left(y_{m}\right)-F_{1}\left(y_{m}\right)\right]}^{2},\\ \theta & =\frac{KL}{{\left(K+L\right)}^{2}}\left(\theta_{1}+\theta_{2}\right), \end{array}$$

where sgn(x) is the sign function. The value of θ gives a measurement of differences between the two CDFs. The magnitude of θ depends on the difference between the cumulative distributions; i.e., if $F_{0}$ and $F_{1}$ differ significantly, |θ| will be large. Moreover, observe that the sign information is crucial to determine whether $F_{1}$ is above or below $F_{0}$, a positive θ indicates that $F_{0}$ is mostly above $F_{1}$, which means that the backoff window values for sensor *m* are larger than expected, and hence, sensor *i* is not doing a backoff attack. The opposite happens when θ sign is negative, indicating that $F_{0}$ is mostly under $F_{1}$, which means that the observed values of backoff window for the sensor *m* are smaller than expected, and hence, a backoff attack is being detected. Hence, the reputation in the MAC layer of each sensor *m* is determined as follows [30]:

(11) $$t_{MAC}=e^{-D^{2}},\;D=\min\left\{\theta ,0\right\}.$$

**Figure 3:**
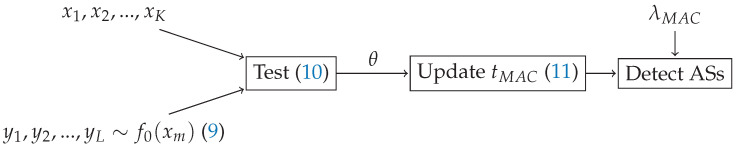
Block diagram for the defense mechanism against the backoff attack. The inputs are a set of *K* backoff windows used by a sensor and a set of *L* backoff window sampled from $f_{0}\left(x_{m}\right)$ (9). In this case, the defense mechanism parameters are *K*, *L* and $\lambda_{MAC}$, and the result is an updated list of sensors detected as ASs.

In (11), $t_{MAC}$ will be 1 (i.e., sensor *m* is completely trusted) when θ is positive, which is the case in which there is no attack. As θ becomes negative, $t_{MAC}$ decreases, indicating that sensor *m* is less trusted because it might be performing a backoff attack. If $t_{MAC}$ falls below a certain threshold $\lambda_{MAC}$, the sensor *m* is considered to be an AS and it is banned from the network. A scheme summarizing the defense mechanism is in Figure 3.

## 4. Deep Reinforcement Learning Attacker Architecture

We start our discussion of the DLA architecture by relating the attack description from Section 3 with the swarm model presented in Section 2. First, let us note that the state of the defense mechanism is the reputation value for each sensor ($t_{PHY}$, $t_{MAC}$). If the ASs had access to this value and knew the reputation threshold $\lambda_{PHY}$ or $\lambda_{MAC}$, we would have an MDP setting where ASs could choose what to do in order not to be discovered following similar procedures to [61]. However, this is not a realistic assumption, as in many cases the ASs may not know the concrete parameters of the defense mechanism, or even the defense technique implemented by the defense mechanism. However, we can assume that ASs have access to values that can be observed, such as the time from last transmission attempt, the energy levels reported or whether the node has been banned from the network. This means that we shift from an MDP model to a POMDP, as ASs only have access to observations rather than the state of the defense mechanism. This also means that ASs do not have a priori knowledge about the defense mechanism. Another important remark is that we may assume that cooperation among ASs may increase the attack performance. As we have mentioned in Section 2, we can model this situation by using the swarm model. Concretely, we can use the swarMDP model, as we can assume that all ASs are homogeneous and interchangeable (i.e., they share a common goal and the set of states, actions, observations and policy), and we may benefit from the computational advantage that this model brings over the Dec-POMDP model.

Thus, let us model our attack situation by making use of the swarMDP model. We assume that we have a swarm of ASs trying to attack a WSN, where GSs are all sensors of the WSN except the ASs. At each timestep *n*, the ith AS has a certain observation $o_{i}^{n}$ (which, as we will see shortly, may include information from other sensors), and it uses its policy function, $\pi_{\theta }\left(o_{i}^{n}\right)$, to select a certain action $a_{i}^{n}$. After this action is executed, the ith AS receives the common reward $r^{n}$ and the next observation, $o_{i}^{n+1}$. Under this scheme, we have the following characteristics for the SSDF attack:

- A continuous set of actions in the range [0,1], where the action indicates the normalized energy that the sensor reports to the FC.
- We consider that the reward to each AS is +1 if the FC decides that there is a primary transmitting, whereas the reward to each AS is 0 if the FC decides that there is no primary transmitting. We use a maximum number of timesteps for each episode and if all ASs are discovered we terminate the episode. The DLA must therefore learn to maximize the number of timesteps without being discovered in order to maximize its reward. The punishment for being detected consists of being banned from the network, which means that the agent stops receiving rewards. Furthermore, the reward also tries to increase the probability of misdetection of the WSN; that is, it makes the FC believe that the primary is transmitting more often than it really does.
- In order to build the observations vector, each agent stores its last five actions (i.e., energy reported) and a binary flag indicating whether the agent has been already discovered by the defense mechanism (and hence, banned from the network) or not. We assume that agent *i* can also access the observations of other sensors j≠i, i.e., ASs can communicate their local observations to other sensors (which can be implemented either by direct communication among nodes or by observing the behavior of other sensors).

For the backoff attack, we have the following:

- The action space is composed by two discrete actions that indicate whether the sensor starts transmitting or not. That is, the actions start transmitting in the current time slot or not. Hence, two consecutive actions may be separated by several physical time slots if no sensor starts transmitting.
- The reward is −1 in case that a GS starts transmitting and 0 otherwise. Our choice of the rewards is different as the attacks have different targets: in case of the SSDF attack, we want to detect the primary as often as possible. However, in the backoff attack, we want GSs to transmit as little as possible. We set a fixed simulation time, which is completed regardless of whether the ASs are discovered. Thus, the DLA needs to learn not to be discovered while preventing GSs from transmitting.
- In order to build the observations vector, we use the time difference between the current timestep and the last *K* transmissions (i.e., this indicates the frequency of attempted transmission of the sensor). This difference is normalized by the maximum number of timesteps. We also add a binary flag indicating whether the agent has been discovered by the defense mechanism or not, as in the SSDF case.

We remark that, in both attacks, the observation does not match the system state, which are the reputation ($t_{PHY}$ and $t_{MAC}$) of each sensor. Rather, each ASs only has access to a limited set of information about its past actions and information about whether a sensor has been banned or not by the defense mechanism. In addition, since we use a model-free DRL approach, and we do not require a model of the transition probabilities for the attacks, which is also a significant advantage over methods that require explicit models of these probabilities, as this allows us to significantly ease the required computational load [31]. Furthermore, we aim to study whether communicating the local observations can be exploited by the DLA agent to learn better attack mechanisms. Therefore, we also compare with the non-communication case.

The set of observations, actions and rewards of each agent in each timestep is used to update the common policy $\pi_{\theta }$ using TRPO. We use as policy a feed-forward DNN, which takes as input the observation vector $o_{i}^{n}$ and outputs the action. The two first layers of the network have 256 neurons and use rectified linear activations. As TRPO can be used for continuous and discrete actions, we use it for both of our attacks, as the SSDF attack has a continuous action set, while the backoff attack has two discrete actions.

**Figure 4:**
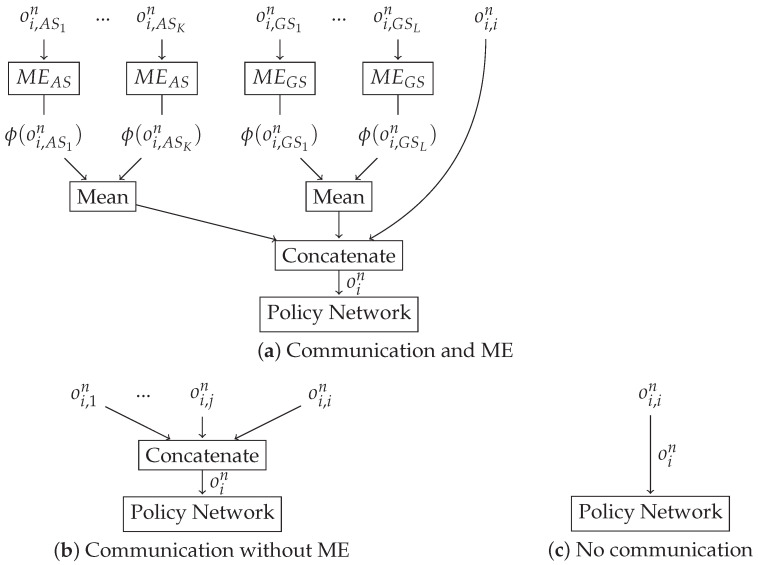
Sketch of the different DLA architectures. The difference in the architectures lies in how the observation $o_{i}^{n}$ is obtained. In (**a**,**b**), there is communication among the swarm agents, and hence, each agent *i* has access to the local observations of the rest of the agents. (**a**) shows the architecture when a Mean Embedding is used: we use separate Mean Embeddings for ASs and GSs, and we assume that there are K+1 ASs (agent *i* is also an AS), *L* GSs and that $o_{i}^{n}$ is the concatenation of the mean values of the Mean Embeddings and the local information of the agent *i*. (**b**) shows the architecture when there is communication but we do not use any Mean Embedding: in this case, $o_{i}^{n}$ is the concatenation of the observations. (**c**) shows the no-communication case in which only the local observation is available.

Finally, as mentioned before, we want to test the influence of communication in the attack performance. As mentioned before, a possible option consists of concatenating the local observation of each sensor, although this approach means that the policy input depends on the number of sensors, hence complicating the learning process when there are several sensors. Moreover, this method is not invariant to the number and order of sensors. A better alternative is MEs, which we use to combine the observations from the agents in the case that there is communication in a meaningful way. We emphasize that MEs allow a combination that is invariant to the number and order of the agents. We remark that there are two different kind of sensors: ASs and GSs. In order to combine the sensor observations meaningfully, we concatenate one mean embedding for the observations of the ASs and another with the observations of the GSs. A graphical description of all the architectures that we use are in Figure 4.

## 5. Results

We evaluate our DLA architectures on the SSDF and the backoff attack on a WSN that contains 10 GSs and {1,3,10} ASs. For each attack, we tested four different setups:

- Communication and Neural Networks Mean Embedding (CNNME) setting: in this case, ASs communicate among them and use MEs to aggregate information (situation (a) in Figure 4). Concretely, we use NNME, which is based on using a DNN as ME where the weights of the DNN are trained together with the TRPO policy.
- Communication and Mean-based Mean Embedding (CMME) setting: in this case, ASs communicate among them and use MEs to aggregate information (situation (a) in Figure 4). Concretely, we use MME, which consists of using the mean operation as ME.
- Communication without using mean embeddings (C) setting: in this case, ASs communicate among them without using any ME to aggregate information (situation (b) in Figure 4). In this case, the input size of the policy network is equal to the dimension of $o_{i}^{n}$ and thus increases with the number of ASs, while it remains invariant for all other cases.
- Without any communication among ASs (NC) setting (situation (c) in Figure 4). In this case, each AS only uses its local observations in order to obtain their local policy.

For CNNME, CMME and C settings, the reward that each agent maximizes is the sum of the rewards of all ASs, by following the swarMDP model (i.e., they have a common goal). We train the policy network until convergence, using 500 TRPO iterations for both attacks. In each iteration, a batch with $10^{4}$ timesteps is used to optimize the policy. For each combination of number of ASs, DLA setup and attack type, we repeat the training using 10 different seeds, as the results of DRL methods are known to be dependent on initial conditions [62]. The code used is available at https://github.com/jparras/dla (accessed on 11 June 2021).

### 5.1. Baselines

We remark that we do not know the optimal solution to the underlying POMDP that models the attack. In order to evaluate the quality of the results of the DLA agent, we compare the results obtained to three baselines policies based on the always-false, always-busy and always-free attack policies from [63]:

- Random policy (RP), which samples the actions uniformly from the action space; i.e., in the SSDF attack, it means that ASs report a random energy level, and in the backoff attack, ASs transmit randomly.
- Always High (AH), which selects the highest action possible; i.e., in the SSDF attack, it means that ASs always report the maximum energy level, and in the backoff attack, ASs always transmit.
- Always Low (AL), which selects the lowest action possible; i.e., in the SSDF attack, it means that ASs always report the minimum energy level, and in the backoff attack, ASs never transmit.

The advantages of these baselines used in the literature are that they are simple to implement, they do not require knowing the defense mechanism and they have a low complexity. However, they are unable to adapt in order to exploit a certain defense mechanism, although, as shown in [63], they can be successful attack policies in some cases. More complex attacks could be devised by having knowledge of the defense mechanism [31], although we do not use them in this paper to have a fair comparison with DLA: none of the policies we test relies on an a priori knowledge of the defense mechanism.

### 5.2. Spectrum Sensing Data Falsification Attack

We use finite horizon episodes; i.e., in each episode, the FC asks the sensors up to 250 times to report the energy level they measure. We consider that the duty cycle is 0.2; i.e., the probability that the channel is actually occupied by a primary transmitter is 0.2. If an AS is detected, the episode ends for this AS, since the FC does not ask it to send more reports. We implement the defense mechanism explained in Section 3.1, with η=1, ζ=1.6 [30] and $\lambda_{PHY}=0.5$. If the reputation of a sensor $t_{PHY}$ falls below $\lambda_{PHY}$, the sensor is detected as an AS and the episode ends. If the AS attacks indiscriminately, the episode will end early and its reward will be low. At each timestep, the defense mechanism is invoked and the sensor reputation is updated.

At the beginning of each episode, we pick the sensor distances to the FC, $d_{m}$, from a uniform random distribution in the range [800,1000] m. We consider that the transmitter power is $P_{tx}=23$ dBm and use the following path loss expression:

(12) $$P_{m}=P_{tx}-\left(35+3\cdot 10\log_{10}\left(d_{m}\right)\right),$$

where $P_{m}$ is the received power in the sensor *m* in dBm. This expression allows us to obtain $SNR_{m}=10^{\frac{P_{m}-NP}{10}}$, where we consider the noise power to be NP=−110 dBm. We consider the time-bandwidth product to be k=5. Finally, we generate the $E_{m}$ values sampling from the distributions in (2), depending on whether the primary is transmitting or not.

### 5.3. Backoff Attack

The backoff attack is simulated for $5\times 10^{5}\;\mu s$. In each timestep, an AS decides whether to start transmitting or stay idle. Hence, timesteps are related to backoff steps, not to physical time (i.e., if an agent starts transmitting in timestep *n*, timestep n+1 will take place when that agent finishes transmitting). We do not penalize collisions. The defense system explained in Section 3.2 is used, with K=5 and L=1000. If the reputation of a sensor $t_{MAC}$ falls below $\lambda_{MAC}=0.5$, the episode ends for this sensor. We note that if the AS attacks indiscriminately, the episode ends early with a low reward: recall that a final reward is given to each AS that corresponds to the remaining reward of the episode; i.e., if the AS is caught, it does not have the opportunity any more to hinder the GSs to transmit, yielding a lower reward. We use the network parameters from Table 1 to simulate the backoff attack. The defense mechanism is run once every give timesteps in order to ease the computational load.

### 5.4. Results

**Figure 5:**
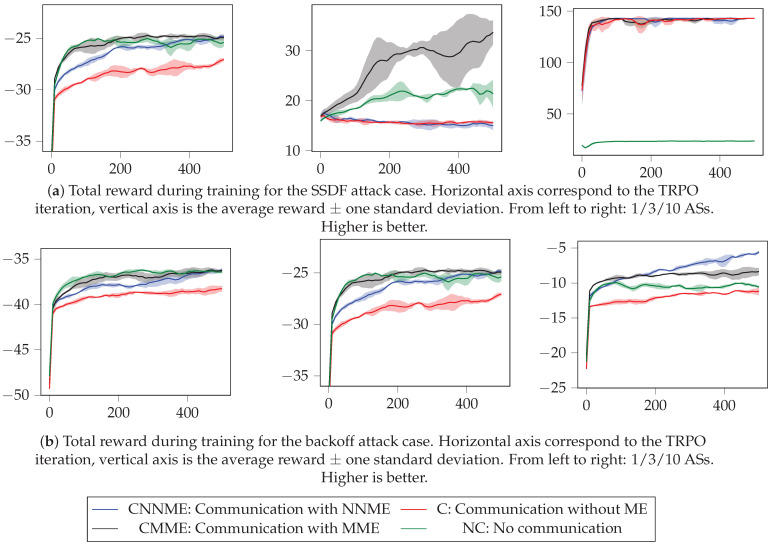
Training results for the SSDF and backoff attacks for the best three seeds. We observe that the variability between seeds is higher in the SSDF attack. Also, note how DLAs improve their reward as training progresses, and specifically, observe that they do so simply by interacting with the defense mechanism.

The results for both attacks can be observed in Figure 5 and Table 2, Table 3 and Table 4. First, in Figure 5, we observe that ASs do learn to successfully exploit the defense mechanism in both attacks, as the reward increases with the TRPO iterations. There is a higher variability among seeds in the SSDF attack, which is a known problem that arises when using DRL methods [62]. The remarkable fact is that this attack improvement is achieved simply by interacting with the defense mechanism, as the ASs do not have a priori knowledge about it.

In Table 2, we show the final rewards obtained by using all the DLA architectures and compare them with the baseline policies. The baselines provide clearly worse results than the DLA architectures proposed. If we focus on the results of the SSDF attack, we first see that DLA with communication and ME have an advantage over the other methods. With a single AS, the performance of all DLA is similar. When there are 10 ASs, the defense mechanism can be overpowered by continuously reporting high energy levels: thus, not only all communication-based DLAs, but also the AH baseline, provide the same rewards. In all cases, all DLAs provide a significantly better reward than the baselines (except for the commented case of AH baseline with 10 ASs).

If we focus on the results of the backoff attack, which has a statistically complex defense mechanism (see Section 3.2), we note, again, that the baseline results are considerably improved by all DLAs. This is due to the fact that DLAs are able to learn to exploit the defense mechanism successfully, while the baselines fail to provide good results. In this case, having communication between agents using an ME consistently provides the best results: we believe that this is due to the fact that the swarMDP model is specially suited for the context of network attacks, as it facilitates exploiting the defense mechanism by using the local observations of the other nodes.

One of the main targets in our DLA was to be able to attack without being discovered, that is, to camouflage. Hence, the rewards presented in Section 4 were designed to exploit the defense mechanism and not be detected. Thus, the reward scheme implicitly induces a trade-off between camouflage and exploit of the defense mechanism that can be observed in Table 3 and Table 4. First, Table 3 shows the detection proportion for each DLA and baseline: in the SSDF case, a low detection is usually related to a high reward (i.e., low detection and attack come together) except for the AL baseline, which we remark consisted of always reporting a low energy level (and hence, ASs are never detected). However, in the backoff attack, the ranges of detection proportion are larger: this may be due to a cooperative behavior among ASs, such that some ASs are detected for the sake of getting a better reward (which, recall, is shared among all ASs).

Table 4 shows the attack results in terms directly related to the attack target. In the SSDF case, we show the proportion of times that the primary is detected, which is always in the 10 ASs case: as we mentioned before, 10 ASs are enough to overpower the defense mechanism in this attack. In the backoff case, we show the bits transmitted by GSs, and there are two conclusions from the results that are important. First, the baselines actually provide GSs with more resources: either the ASs are detected fast (RP, AH baselines in Table 3) and hence banned from the network, or they do not attack (AL baseline), and both situations lead to the network to have less sensors to split the bandwidth, as mostly GSs are transmitting. For instance, in the 10 ASs case, there are 10 GSs to access the bandwidth, as 10 ASs are either banned or not transmitting, compared to 20 sensors if all the sensors were GSs. Second, the DLA may significantly reduce the transmission rates of GSs, up to an 80% in the case of the CNNME with 10 ASs, which is a very notable decrease in the system throughput.

## 6. Conclusions

In this work, we propose using DRL tools in order to create an attacker architecture able to challenge several defense mechanisms used in WSN. We considered attacks on the PHY and MAC layers, and show that our approach poses strong challenges to current defense mechanisms:

1. We do not need to know the state of the system (i.e., the reputations), as DLA relies only on partial observations (i.e., information about how the sensors have interacted in the past that can be observed by other sensors). Even though we have only partial observations, they provide very good results, especially when the ASs are able to communicate their observations—in Table 2, the results using communication and ME consistently are the best. However, even when this communication is not considered—i.e., the NC setup—the results are still better than the baselines. Even though the underlying model of the attack is a POMDP, DLA learns to attack having only a limited amount of past observations. Thus, when attacking a network, if communication among ASs is possible, our results point towards using ME to aggregate the local information in a meaningful way. We test using two different ME types: NNME, which may extract better features at the cost of extra training complexity, and MMEs, which provide a lower training computational cost but obtain more limited features for the policy. The results shown in Table 2 show that both MEs provide good results, and hence, choosing between them may depend on the concrete problem to solve and the training restrictions we may have.
2. Since DLA does not need to know a priori which defense mechanism it is facing, it is a very flexible approach. Thus, DLA could be the base for a universal attacker, successful in exploiting many defense mechanisms. Indeed, DRL methods have been used to provide human-like performance in many Atari games [5], so DRL could also be applied to exploit defense mechanisms in a universal fashion.
3. It is a method with balanced computational requirements. The training process is the most computationally expensive part of the system. However, most of this cost was used in generating samples from the defense mechanisms: training the DNNs using gradient techniques was fast. This low training cost appears because we use simple DNNs, which, however, are enough to exploit the defense mechanisms. Once the DNN is trained, the policy is quick to execute and can be deployed in devices with low computational capabilities.
4. We remark that we have used the same set of hyper-parameters for all of our simulations. We have done no fine-tuning of these hyper-parameters, and thus, our approach may suit very different attack situations with minimal tuning. Equivalently, the results obtained could be improved by doing a fine-tuning for each situation.

However, there are also several drawbacks that arise from the results of this paper and that are future lines of research that can further advance this work:

1. The reward scheme has a strong influence on the attack that is learned. There is a tradeoff between attacking and not being discovered, and hence, modifying the reward scheme will cause the DLA to learn a different attack strategy. In other words, we must carefully design the reward depending on the attack result desired. Note that this is not something specific to our problem but a general problem that arises in the RL field.
2. Our approach relies on the DLA being able to interact with a network continuously, episode after episode. As we considered that ASs could be banned during episodes, this means that the banning must be temporal. If it is permanent, then the DLA could not learn. Note, however, that if ASs had access to a simulator of the defense mechanism, this problem would be overcome.
3. Our DLA is not sample-efficient, as it requires many samples to learn; in addition, the results are dependent on the initial parameters of the policy DNN. These are known problems of DRL methods, which are subject to current research [62]. A very promising research field that could address these problems is few-shot learning, focused on learning from a few samples. There are several works in this area that may be used to improve the sample efficiency of DRL methods, such as [64,65], and hence could further improve the results of attacks to WSN.
4. A related challenge comes from the fact that we have assumed a static defense mechanism, but it could be dynamic and change after a series of attack attempts have been detected. DLA may be currently vulnerable to this defense strategy due to its low sample efficiency, and hence, we expect that the research on increasing the DRL sample efficiency would also be useful to prevent and/or adapt quickly to changes in the defense mechanism. Another alternative could consist in replacing a banned AS with a new one that is initialized based on the experience of the banned AS (i.e., by having the same policy as the banned one).

Hence, the attacking approach that we propose in this work presents strong challenges to current WSN defense mechanisms. First, because of the growing computational capabilities of current hardware, there could soon, if not already, be sensors with enough computational capabilities to implement a DLA [66]. An alternative approach could be based on the use of evolutionary techniques to perform the optimization, as there are many swarm algorithms based on these ideas such as [33,34,35,36]. Second, because DLA is adaptive and flexible, not requiring an a priori modeling of the defense mechanism nor knowledge of their parameters, it can learn to exploit a wide range of defense mechanisms. Thus, it is of capital importance to research defense mechanisms against such attack mechanisms, in order to minimize the threat they pose. A promising defense mechanism could be one in which the defense mechanism also uses RL tools for learning how to defend, which could mean entering the field of Multi Agent Competitive Learning [67,68], which still poses strong challenges.

## Figures and Tables

**Table 1:** MAC network parameters.

Parameter	Value	Parameter	Value
MAC header	272 bits	PHY header	128 bits
ACK	112 bits + PHY header	Bit rate $R_{b,t}$	1 Mbps
SIFS	28 μs	DIFS	128 μs
$T_{\delta }$	1 μs	$T_{p}$	4096 μs

**Table 2:** Final rewards obtained for each combination of attack, number of ASs and setup. The values were obtained averaging 50 episodes for the best seed of each attack policy after training. We show the average reward ± one standard deviation. Bold entries show the best results, where a Welch test is used to detect whether means are significantly different for a significance level α=0.01. Higher is better.

	ASs	CNNME	CMME	C	NC	RP	AH	AL
SSDF	1	22.66±3.36	22.07±4.54	19.93±4.92	22.79±4.87	5.35	0.30	11.66
	3	18.02±6.02	36.22±10.51	17.4±6.03	22.95±3.33	14.47	1.42	0.53
	10	142.88±0.00	142.88±0.00	142.88±0.00	24.97±6.78	23.06	142.88	0.00
Backoff	1	−35.86±4.97	−35.54±3.96	−37.51±3.46	−34.90±3.30	−75.88	−77.81	−44.62
	3	−24.48±3.74	−24.36±3.47	−26.72±3.68	−24.7±3.21	−71.40	−78.84	−43.67
	10	−4.38±3.10	−7.54±2.49	−10.39±3.10	−9.12±2.92	−66.43	−78.16	−43.99

**Table 3:** Proportion of agents detected as ASs by the defense mechanism for each combination of attack, number of ASs and setup. The values were obtained averaging 50 episodes for the best seed of each policy after training. We show the average proportion ± one standard deviation.

	ASs	CNNME	CMME	C	NC	RP	AH	AL
SSDF	1	0.00±0.00	2.00±14.00	2.00±14.00	2.00±14.00	82.00	100.00	0.00
	3	53.33±25.82	0.00±0.00	54.00±24.85	22.00±20.67	77.33	100.00	0.00
	10	0.00±0.00	0.00±0.00	0.00±0.00	61.00±21.93	80.60	0.00	0.00
Backoff	1	22.00±41.42	4.00±19.60	42.00±49.36	8.00±27.13	100.00	100.00	0.00
	3	47.33±35.96	30.00±34.80	41.33±28.72	21.33±21.87	100.00	100.00	0.00
	10	66.80±18.16	59.20±26.97	74.00±18.00	40.60±21.58	100.00	100.00	0.00

**Table 4:** Final results in terms of primary detection percentage (SSDF) and kbits that GSs transmit (backoff), obtained for each combination of attack, number of ASs and setup. The values were obtained averaging 50 episodes for the best seed of each attack policy after training. We show the average value ± one standard deviation. Higher is better in SSDF; lower is better in backoff attack.

	ASs	CNNME	CMME	C	NC	RP	AH	AL	No ASs
SSDF	1	15.91±2.22	15.34±3.16	14.65±3.40	16.06±3.26	9.84	10.80	8.24	8.00
	3	14.61±12.68	25.75±7.36	12.03±4.14	16.58±2.23	15.78	45.80	0.35	28.00
	10	100.00±0.00	100.00±0.00	100.00±0.00	17.16±4.42	18.09	100.00	0.00	22.00
Backoff	1	258.79±19.43	258.46±17.36	275.01±15.78	252.81±16.83	328.83	326.78	350.13	255.4
	3	167.03±24.65	160.65±19.23	182.85±26.77	161.46±17.51	312.20	331.12	348.00	211.87
	10	27.53±23.69	43.50±16.54	60.87±17.84	54.07±20.50	295.65	328.25	348.73	130.57

## Abbreviations

The following abbreviations are used in this manuscript:

A3C	Asynchronous Advantage Actor Critic
ACER	Actor Critic with Experience Replay
ACK	Acknowledgement
AH	Always High
AL	Always Low
AS	Attacking Sensor
CDF	Cumulative Distribution Function
CM	Cramer-von Mises
CNNME	Communication and Neural Networks Mean Embedding
CMME	Communication and Mean-based Mean Embedding
DLA	Deep Learning Attacker
DNN	Deep Neural Network
DoS	Denial of Service
DRL	Deep Reinforcement Learning
DRQN	Deep Recurrent Q-Networks
DQN	Deep Q-Networks
FC	Fusion Center
GS	Good Sensor
MAC	Medium Access Control
MDP	Markov Decision Process
ME	Mean Embedding
MME	Mean-based Mean Embedding
NNME	Neural Networks Mean Embedding
PHY	Physical
POMDP	Partially Observable Markov Decision Process
PPO	Proximal Policy Optimization
QoS	Quality of Service
RL	Reinforcement Learning
RNN	Recurrent Neural Network
RP	Random Policy
SNR	Signal-to-Noise Ratio
SSDF	Spectrum Sensing Data Falsification
TRPO	Trust Region Policy Optimization
WSN	Wireless Sensor Network
